# 2623. Persistence of SARS-CoV-2 S IgG Antibodies in Children that suffered from COVID-19 and Association of Antibody Levels with Vaccination Status

**DOI:** 10.1093/ofid/ofad500.2236

**Published:** 2023-11-27

**Authors:** Federico Di Gregorio, María S Tineo, Florencia A Bonnin, Lidia Torrado, María F Pavan, Lorena I Ibañez, Analía Toledano, Ana Caratozzolo, Patricio L Acosta, Maria M Contrini, Laura Talarico, Eduardo L López

**Affiliations:** Hospital de Niños Ricardo Gutierrez, Buenos Aires, Ciudad Autonoma de Buenos Aires, Argentina; Hospital de Niños Ricardo Gutierrez, Buenos Aires, Ciudad Autonoma de Buenos Aires, Argentina; Pediatric Infectious Disease Program, Hospital de Niños Ricardo Gutiérrez, Universidad de Buenos Aires, Buenos Aires, Ciudad Autonoma de Buenos Aires, Argentina; Hospital de Niños Ricardo Gutierrez, Buenos Aires, Ciudad Autonoma de Buenos Aires, Argentina; INQUIMAE, Buenos Aires, Buenos Aires, Argentina; INQUIMAE, Buenos Aires, Buenos Aires, Argentina; Pediatric Infectious Diseases Program, Hospital de Niños "Dr. Ricardo Gutiérrez", Universidad de Buenos Aires, Buenos Aires, Ciudad Autonoma de Buenos Aires, Argentina; Pediatric Infectious Disease Program, Hospital de Niños Ricardo Gutiérrez, Universidad de Buenos Aires, Buenos Aires, Ciudad Autonoma de Buenos Aires, Argentina; Pediatric Infectious Disease Program, Hospital de Niños Ricardo Gutiérrez, Universidad de Buenos Aires, Buenos Aires, Ciudad Autonoma de Buenos Aires, Argentina; Hospital de Niños Ricardo Gutierrez, Buenos Aires, Ciudad Autonoma de Buenos Aires, Argentina; Hospital de Niños Ricardo Gutierrez, Buenos Aires, Ciudad Autonoma de Buenos Aires, Argentina; Pediatric Infectious Disease Program, Hospital de Niños Ricardo Gutiérrez, Universidad de Buenos Aires, Buenos Aires, Ciudad Autonoma de Buenos Aires, Argentina

## Abstract

**Background:**

Serum levels of IgG antibodies against SARS-CoV-2 spike protein (S) in children who suffered COVID-19 have been found to last between 4 to 12 months. We aimed to characterize the antibody response against SARS-CoV-2 after 1 year of COVID-19 in unvaccinated and vaccinated children.

**Methods:**

A cohort study included unvaccinated and vaccinated children (< 18 years) with a previous SARS-CoV-2 infection. Blood samples were collected during the acute phase, in convalescence and 1 year after suffering the disease. For comparative purposes, vaccinated adult relatives who suffered COVID-19 in the same period were included. SARS-CoV-2 S IgG serum levels were determined by immunoassay. Neutralizing activity was determined by the decrease of GFP expression in SARS-CoV-2 S-pseudotyped lentivirus infected HEK-293T cells.

**Results:**

Eighty-four children were included, median age 8 years (IQR 2-12), 54% (n=45) were females; 38% (n=32) were vaccinated. Also, 50 adults were studied, median age 35 years (IQR 29-44), 76% (n=38) were females. SARS-CoV-2 S IgG levels 1 year after COVID-19 decreased 2.2- to 10.8- fold compared to convalescent levels in unvaccinated children. SARS-CoV-2 S IgG titers positively correlated with neutralizing antibody titers in convalescence and 1 year after COVID-19 (p< 0.05). SARS-CoV-2 S IgG levels were significantly higher in children with incomplete (p=0.0088) and complete/booster (p< 0.0001) vaccination as compared to unvaccinated children. Children that received mRNA vaccines showed higher SARS-CoV-2 IgG titers than children that received an inactivated vaccine (p< 0.0001). SARS-CoV-2 S IgG titers positively correlated with age in vaccinated children (r_S_=0.4303, p=0.014). SARS-CoV-2 IgG titers 1 year after COVID-19 did not associate with disease severity or the presence of comorbidities. No significant differences in SARS-CoV-2 S IgG levels were found between vaccinated children and adults, at the same vaccination level.

**Conclusion:**

These findings highlight the importance of child vaccination to maintain a long-term humoral immune response against SARS-CoV-2 despite having been infected. Also, mRNA vaccines elicited a higher antibody response than inactivated vaccines against SARS-CoV-2 in children.

**Disclosures:**

**All Authors**: No reported disclosures

